# Supplementary figures and images for: Association between abdominal obesity and diabetic retinopathy in patients with diabetes mellitus: A systematic review and meta-analysis
Source: PLoS One. 2023 Jan 5;18(1):e0279734. doi: 10.1371/journal.pone.0279734 (PMC9815584; doi:10.1371/journal.pone.0279734)

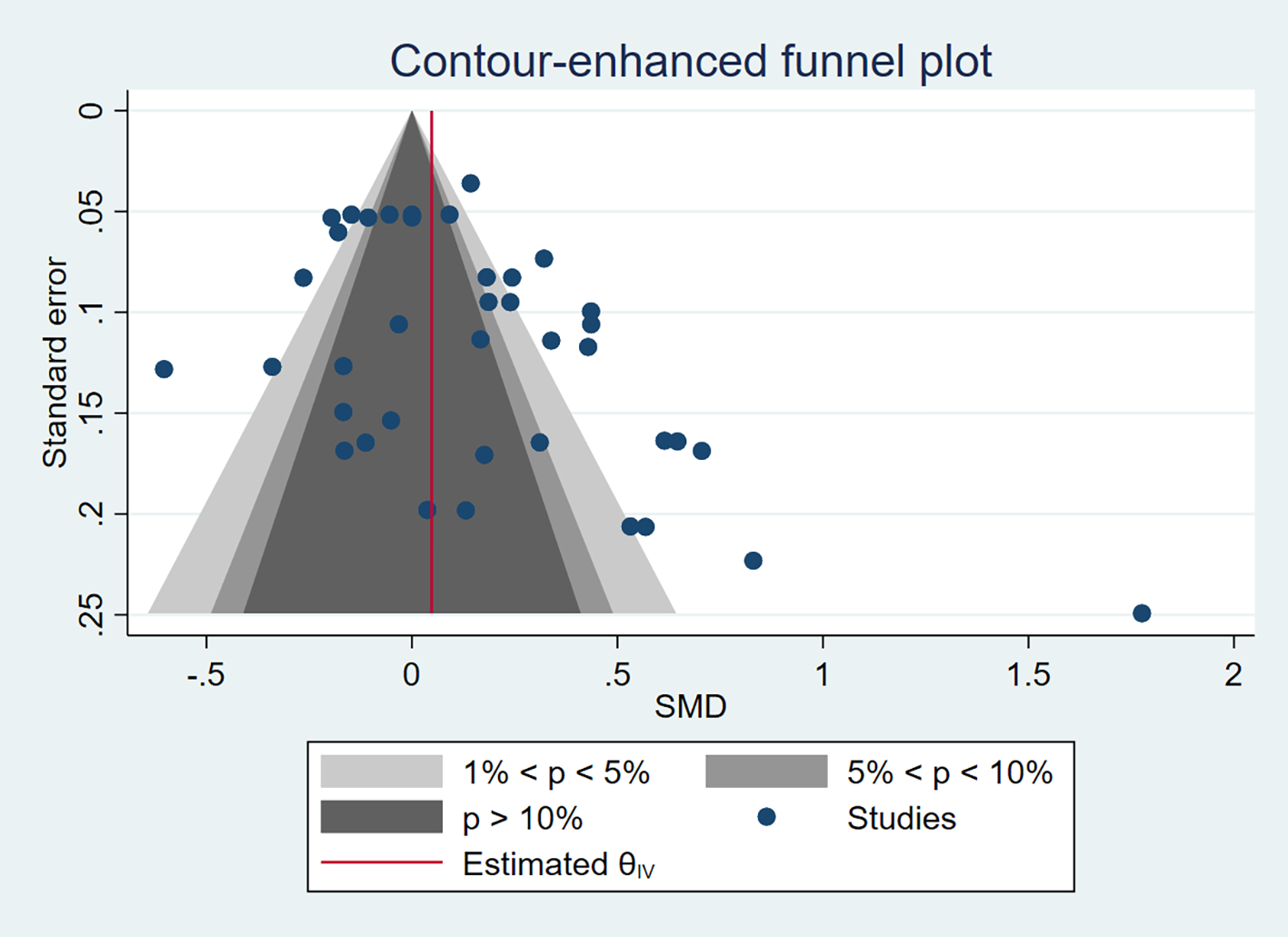

Supplement: S1 Fig — Abbreviations: SMD, standardised mean differences; P, probability value. (TIF) [file pone.0279734.s002.tif]

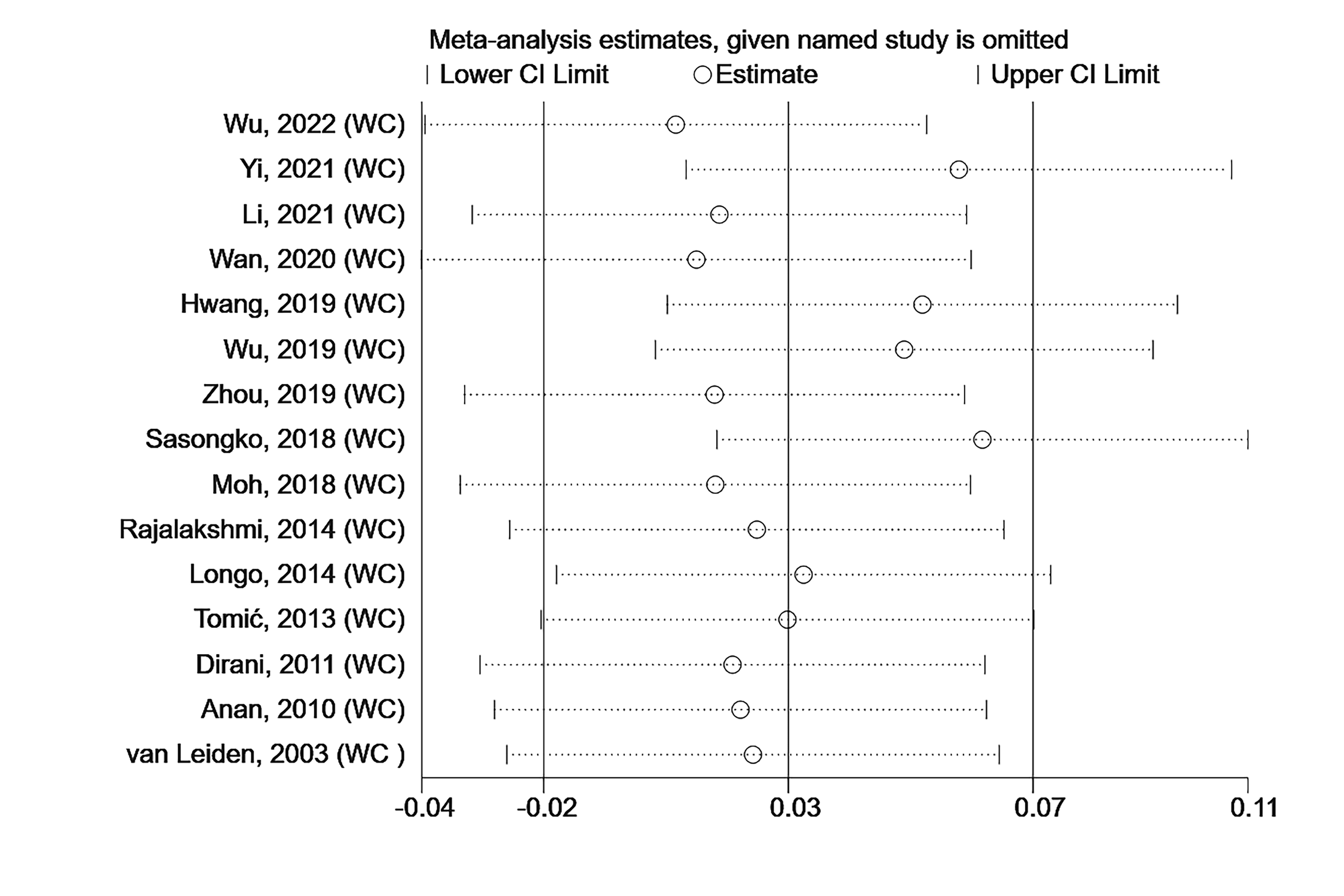

Supplement: S2 Fig — Abbreviations: WC, waist circumference; CI, confidence intervals. (TIF) [file pone.0279734.s003.tif]

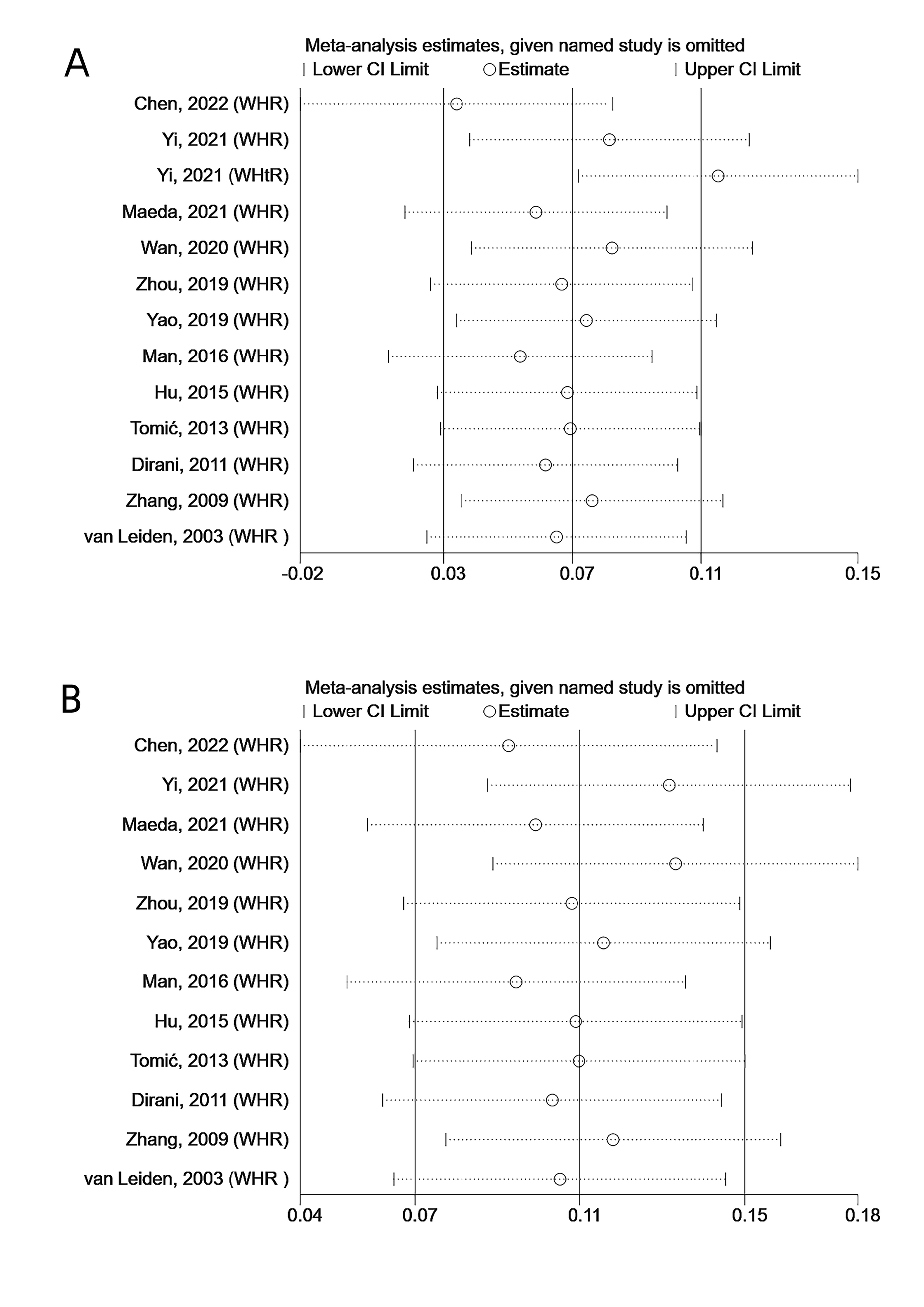

Supplement: S3 Fig — (A) Sensitivity analysis including WHR and WHtR. (B) Sensitivity analysis of subgroup after excluding WHtR. Abbreviations: WHR, waist-hip ratio; WHtR, waist-height ratio; CI, confidence intervals. (TIF) [file pone.0279734.s004.tif]

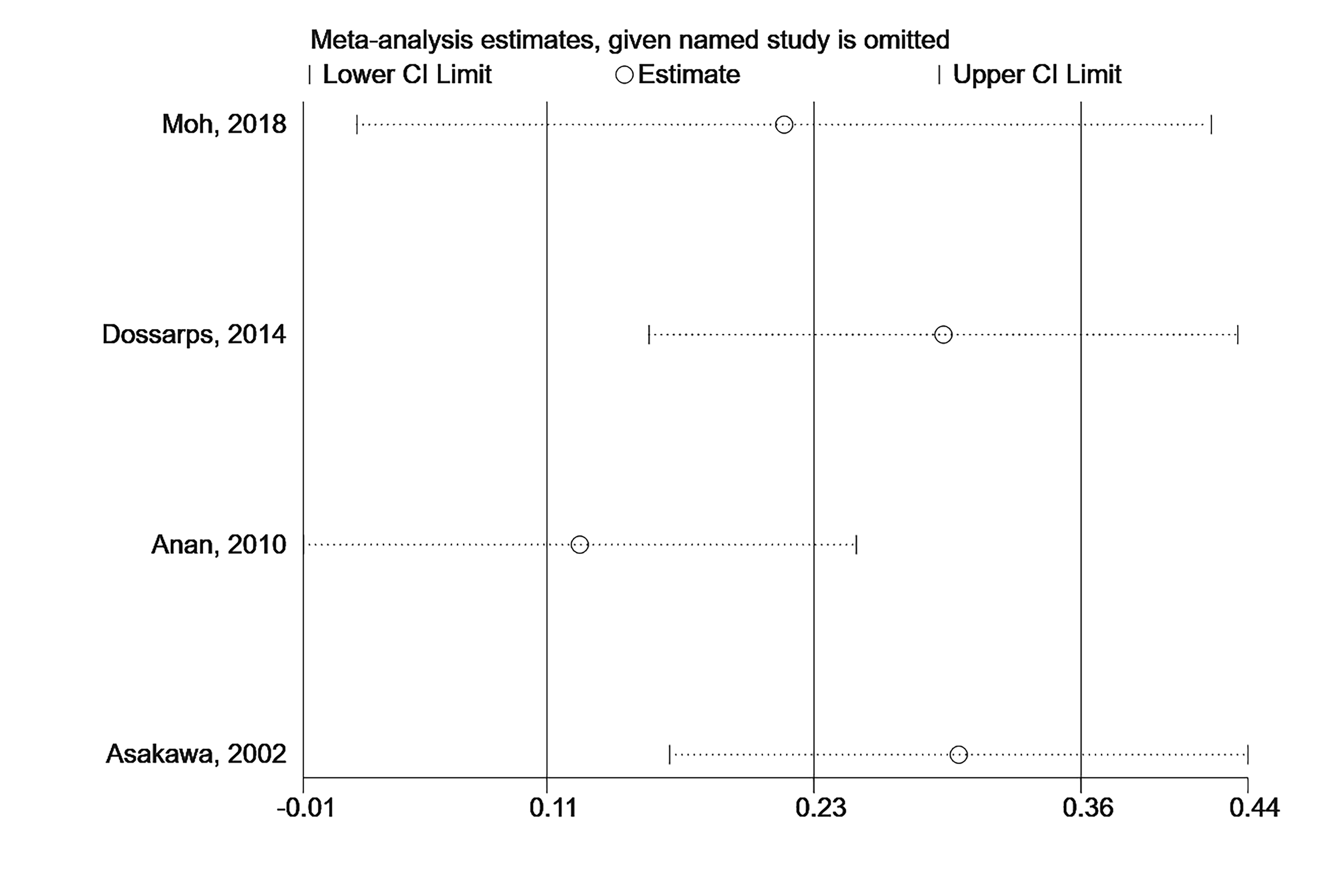

Supplement: S4 Fig — Abbreviations: VFA, visceral fat area; CI, confidence intervals. (TIF) [file pone.0279734.s005.tif]

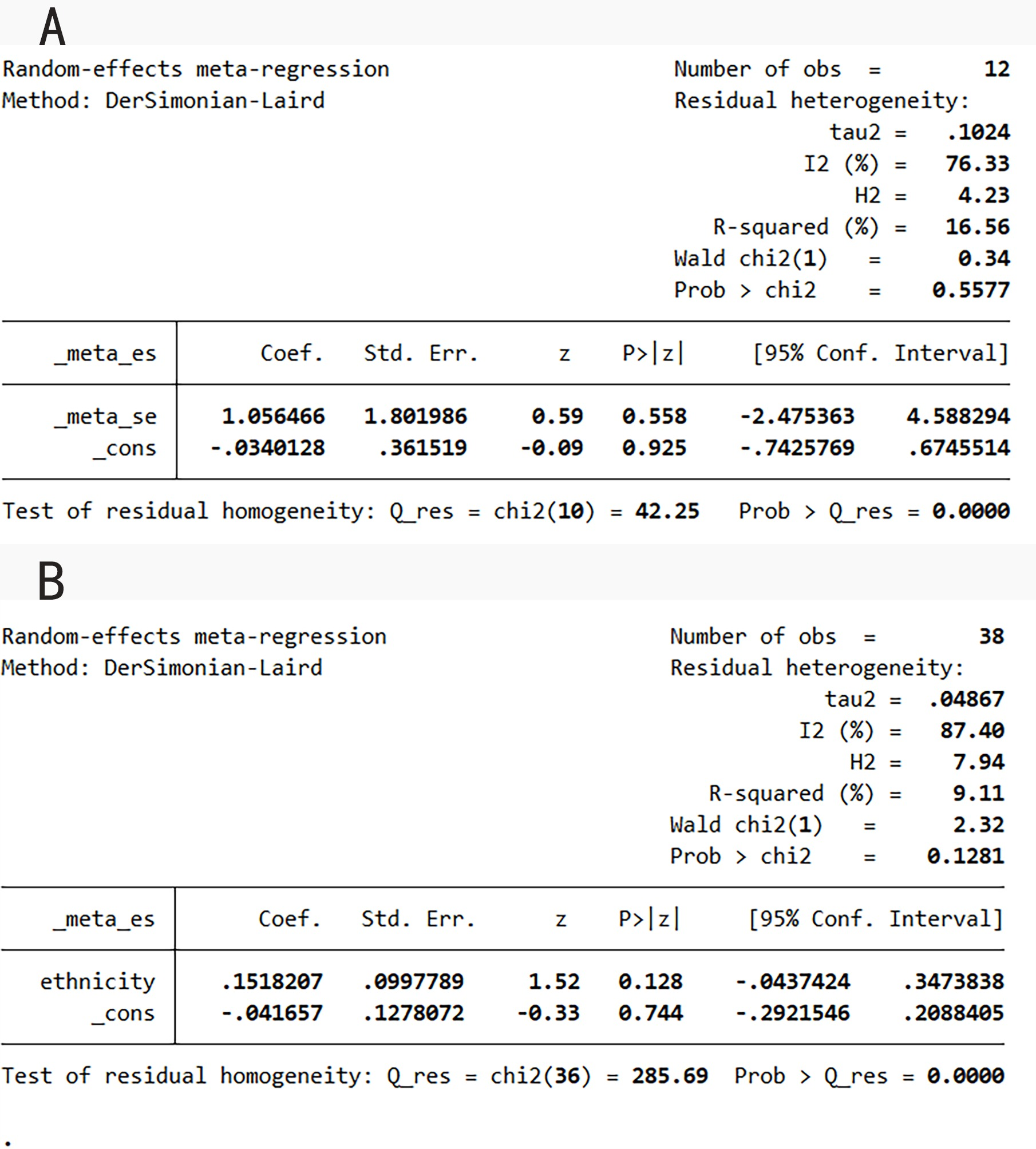

Supplement: S5 Fig — (A) meta-regression analysis of DR severity. (B) meta-regression analysis of ethnicity. (TIF) [file pone.0279734.s006.tif]
