# Supplementary material for: Association between abdominal obesity and diabetic retinopathy in patients with diabetes mellitus: A systematic review and meta-analysis
Source: PLoS One. 2023 Jan 5;18(1):e0279734. doi: 10.1371/journal.pone.0279734 (PMC9815584; doi:10.1371/journal.pone.0279734)
Supplement: S1 Table — (DOCX) [file pone.0279734.s007.docx]

| **(**Obesity, Abdominal[MeSH Terms] OR abdominal obesity[Title/Abstract] OR abdominal fat[Title/Abstract] OR abdominal adiposity[Title/Abstract] OR central obesity[Title/Abstract] OR visceral fat[Title/Abstract] OR visceral obesity[Title/Abstract] OR visceral adiposity[Title/Abstract] OR visceral adipose tissue[Title/Abstract] OR anthropometry[Title/Abstract] OR waist circumference[Title/Abstract] OR waist size[Title/Abstract] OR waist hip ratio[Title/Abstract] OR waist height ratio[Title/Abstract] OR waist[Title/Abstract] OR WC[Title/Abstract] OR WHR[Title/Abstract] OR WHtR[Title/Abstract] OR VFA[Title/Abstract]**)** |
| --- |
| **AND** |
| **((**Diabetic Retinopathy[MeSH Terms] OR Diabetic Retinopathies[Title/Abstract] OR Retinopathies, Diabetic[Title/Abstract] OR Retinopathy, Diabetic[Title/Abstract] OR diabetic eye diseases[Title/Abstract] OR diabetic macular edema[Title/Abstract]**) OR ((**Diabetes Mellitus[MeSH Terms] OR diabetes[Title/Abstract] OR diabetic[Title/Abstract] OR DM[Title/Abstract] OR T2DM[Title/Abstract] OR T1DM[Title/Abstract] OR Hyperglycemia[Title/Abstract]**)** **AND (**optical coherence tomography[Title/Abstract] OR retinal photographs[Title/Abstract] OR retinal diseases[Title/Abstract] OR microangiopathy[Title/Abstract] OR retina[Title/Abstract] OR fundus[Title/Abstract] OR eye ground[Title/Abstract]**)))** |

**S1 Table Search strategy of Pubmed database**
