# Supplementary material for: Association between abdominal obesity and diabetic retinopathy in patients with diabetes mellitus: A systematic review and meta-analysis
Source: PLoS One. 2023 Jan 5;18(1):e0279734. doi: 10.1371/journal.pone.0279734 (PMC9815584; doi:10.1371/journal.pone.0279734)
Supplement: S2 Table — (DOCX) [file pone.0279734.s008.docx]

| **Study** | **1** | **2** | **3** | **4** | **5** | **6** | **7** | **8** | **9** | **10** | **11** |
| --- | --- | --- | --- | --- | --- | --- | --- | --- | --- | --- | --- |
| Wu, 2022 | √ | √ | √ | ？ | × | √ | √ | √ | √ | √ | √ |
| Yi, 2021 | √ | √ | √ | ？ | √ | × | √ | √ | √ | √ | ？ |
| Li, 2021 | √ | √ | √ | ？ | √ | √ | √ | √ | √ | √ | ？ |
| Wan, 2020 | √ | × | × | ？ | √ | √ | √ | √ | √ | √ | ？ |
| Hwang, 2019 | √ | × | √ | √ | √ | √ | √ | √ | √ | √ | ？ |
| Wu, 2019 | √ | × | √ | ？ | √ | × | √ | √ | √ | √ | ？ |
| Yao, 2019 | √ | × | √ | ？ | × | √ | √ | × | × | √ | ？ |
| Sasongko, 2018 | √ | √ | × | √ | √ | √ | √ | √ | √ | √ | ？ |
| Moh, 2018 | √ | √ | √ | ？ | √ | √ | √ | √ | × | √ | √ |
| Man, 2016 | √ | × | √ | ？ | √ | × | √ | √ | √ | √ | ？ |
| Hu, 2015 | √ | × | √ | × | √ | √ | √ | √ | √ | √ | ？ |
| Rajalakshmi, 2014 | √ | × | √ | ？ | √ | × | √ | × | √ | √ | ？ |
| Dossarps, 2014 | √ | √ | √ | × | √ | × | × | × | √ | √ | ？ |
| Tomić, 2013 | √ | √ | × | ？ | × | √ | √ | √ | √ | √ | ？ |
| Dirani, 2011 | √ | √ | × | ？ | × | √ | × | √ | √ | √ | ？ |
| Anan, 2010 | √ | √ | √ | ？ | × | √ | √ | √ | √ | √ | ？ |
| Zhang, 2009 | √ | × | √ | ？ | × | √ | × | × | √ | √ | ？ |
| van Leiden, 2003 | √ | √ | √ | ？ | √ | √ | √ | √ | √ | √ | √ |
| Asakawa, 2002 | √ | × | √ | ？ | √ | × | × | × | √ | √ | ？ |
| Chaturvedi, 2001 | √ | √ | √ | ？ | √ | √ | √ | √ | √ | √ | √ |
| 1–11 represents the 11 items of the AHRQ recommended criteria “√” means “yes”, “×” means “no”, “?” means “unclear” | | | | | | | | | | | |

**S2 Table Quality assessment of cross-sectional studies according to AHRQ recommended crite**
