# Supplementary material for: Association between abdominal obesity and diabetic retinopathy in patients with diabetes mellitus: A systematic review and meta-analysis
Source: PLoS One. 2023 Jan 5;18(1):e0279734. doi: 10.1371/journal.pone.0279734 (PMC9815584; doi:10.1371/journal.pone.0279734)
Supplement: S3 Table — (DOCX) [file pone.0279734.s009.docx]

| **Study** | **Selection** | | | | **Comparability** | **Exposure** | | | **Total score** | **Quality rating** |
| --- | --- | --- | --- | --- | --- | --- | --- | --- | --- | --- |
|  | **Case definition** | **Representativeness of the Cases** | **Selection of Controls** | **Definition of Controls** | **Comparability of cases and controls** | **Ascertainment of exposure** | **Same method of ascertainment for cases and controls** | **Non-response rate** |  |  |
| Chen, 2022 | * |  | * | * | ** | * | * | * | 8 | Good |
| Maeda, 2021 | * |  |  | * |  | * | * |  | 4 | Fair |
| Zhou, 2019 | * |  |  | * | ** | * | * | * | 7 | Good |
| Longo, 2014 | * |  | * | * | ** | * | * | * | 8 | Good |

**Table S3 Quality assessment of case-control and cohort studies according to NOS.**
